# Deposition of Zinc–Cerium Coatings from Deep Eutectic Ionic Liquids

**DOI:** 10.3390/ma11102035

**Published:** 2018-10-19

**Authors:** Miguel Marín-Sánchez, Elena Gracia-Escosa, Ana Conde, Carlos Palacio, Ignacio García

**Affiliations:** 1 Department of Surface Engineering, Corrosion and Durability, National Center for Metallurgical Research CENIM-CSIC, Av. Gregorio del Amo 8, 28040 Madrid, Spain; mgl.marin.sanchez@gmail.com (M.M.-S.); graciaesc@gmail.com (E.G.-E.); a.conde@cenim.csic.es (A.C.); 2 Department of Applied Physics, College of Science, Module 12, Autonomous University of Madrid, Cantoblanco, 28049 Madrid, Spain; carlos.palacio@uam.es

**Keywords:** zinc, steel, corrosion, ionic liquids, deep eutectic solvents, cerium oxide

## Abstract

This work studies the electrodeposition of zinc and cerium species on carbon steel substrates from choline chloride-based ionic liquid bath in order to develop a protective coating with anti-corrosion, sacrificial, and self-repairing properties. Hull cell tests were used to study the influence of the current density on composition of the coatings and their morphology. Surface morphology, chemical composition and oxidation state of the obtained coatings were examined by scanning electron microscopy (SEM), Energy Dispersive X-ray spectroscopy (EDX), and X-ray photoelectron spectroscopy (XPS), respectively. Furthermore, electrochemical characterization and corrosion tests were performed in order to evaluate the corrosion properties of the electrodeposited Zn–Ce coatings. The cathodic deposition of Zn–Ce was achieved for the first time using the deep eutectic solvent choline chloride-urea as an electrolyte. Cerium was incorporated in the coating as oxide or mixed oxide within the Zn metal matrix. The composition and morphology of the electrodeposited coating were dependent on the applied current density. Electrochemical corrosion tests showed similar corrosion rates for all the coatings. Nevertheless on scratched tests with a ratio area of 15:1, for Zn–Ce coatings cerium oxide somehow migrates from the coating to the high pH cathodic areas developed on the surface of the bare steel substrate. Further study is still necessary to improve the corrosion protection of the Zn–Ce coating for carbon steel.

## 1. Introduction

Cadmium coating has been widely used for corrosion protection of steels in industrial applications. However, its toxic nature makes its use unsustainable, thus requiring an alternative coating with similar protective properties to cadmium [1,2]. Zinc coatings and their alloys [3,4,5,6] are one of the most promising alternatives. Like cadmium, Zn coatings behave as a sacrificial coating. Zn has a more negative corrosion potential than steel, therefore protects the steel substrate by dissolving preferentially in the event of coating damages. However, its higher corrosion rate than cadmium requires a thicker coating to provide similar corrosion protection.

This is the reason why there are still many ongoing studies to developed Zn alloys with lower dissolution rates and improved anti-corrosive properties. Different approaches have been used to deal with this drawback. One of the most common has been focused on the development of a variety of zinc alloys that contain small amounts of other elements to decrease the corrosion kinetics while retain the cathodic potential to the steel. For example, various zinc alloys have been developed and studied with manganese [7], nickel [1], cobalt [8], or iron [9], along with many others [10,11,12].

On the other hand, cerium compounds provide the substrate with an active corrosion protection due to the fact that they act as cathodic inhibitors. In aqueous solution, the cerium inhibition mechanism is based on the blocking of cathodic sites through the precipitation of insoluble cerium oxides/hydroxides. This occurs in areas with high concentrations of OH^-^ ions, where pH increases locally. These compounds inhibit the cathodic reaction, thus reducing the corrosion rate and the anodic reaction [13]. Different methods have been used for the incorporation of cerium-based compounds into metals: by alloying [14], immersion [15], ion implantation [16], sol-gel process [17], vapor deposition [18], and electrodeposition [19,20,21,22], among others. For decades, the electrodeposition process has been widely used because it allows the control of the coating characteristics (surface morphology, thickness, crystalline state, etc.) as well as it is also a low cost process. Cerium oxide can be electrodeposited, both anodic and cathodically. Anodically, the electrodeposited oxide shows as stoichiometry closer to cerium oxide (IV) [23,24], while cathodic electrodeposition produces a mixture Ce (III)/Ce (IV) oxides [25,26,27]. The electrodeposition of cerium, like any other element, can be carried out from different electrolytes such as molten salts, organic or aqueous solutions [28]. These electrolytes have varying disadvantages such as the need for high processing temperatures in the molten salts, or the instability associated with volatile compounds. In addition, the hydrogen evolution in aqueous solutions reduces the efficiency of the cathodic reduction reaction and also prevents the use on high strength steel due to the risk of hydrogen embrittlement [29].

To elude those problems, the use of ionic liquids (ILs) for the electrodeposition of metals and alloys is an excellent alternative to conventional aqueous baths [7,30,31], as well as for cerium species [32,33]. Ionic liquids are potential solvents for metal salts and have a wide electrochemical window, a high thermal and chemical stability, a low vapor pressure and an environmentally friendly behavior, among other characteristics [34]. In 2003, Abbott et al. [35] reported the advantageous properties of a new class of solvents, named Deep Eutectic Solvents (DESs). Since then, these have acquired great importance as they present physical properties similar to the room temperature ionic liquids [35,36]. In fact, DESs are considered to be the fourth generation of ionic liquids even though they are not completely composed of ionic species. DESs are the combination of a hydrogen bond donor (HBD) and hydrogen bond acceptor molecules (HBA) with their synthesis being a quick and simple process [37,38]. In addition, the starting compounds usually have a low price coupled with low harmfulness and high biodegradability [39]. DES based on choline chloride and urea, glycerol or ethylene glycol are the most widely used in a wide range of applications including electrodeposition [12,36,40,41,42,43,44,45].

In this work, cathodic electrodeposition from a deep eutectic ionic liquid was performed in order to obtain the co-deposition of zinc and cerium species. The purpose is to evaluate the potential substitution of cadmium coatings as sacrificial anodes by zinc and the cerium ceramides coating with self-repairing effects.

## 2. Materials and Methods 

### 2.1. Preparation of the Ionic Liquid and Dissolution of the Salts

The DES ionic liquid was prepared by mixing urea (Merck KGaA, Darmstadt, Germany) and choline chloride (ChCl) (Merck KGaA, Darmstadt, Germany) in a 2:1 molar ratio at a temperature of 80 °C with continuous vigorous magnetic stirring until a homogeneous colorless liquid was formed. After this step, the incorporation of the cerium and zinc species to the electrolyte was made by the addition of the ZnCl_2_ and CeCl_3_·7H_2_O salts separately (0.3 M in each case) and together (Zn:Ce ratio 3:1). In all cases, the solution was dried overnight at 80 °C under vacuum and stored under moisture-free conditions before electrodeposition. Remaining moisture was measured by Karl Fisher titration showing a volumetric water content between 0.16 ± 0.09% in ChCl–urea–ZnCl_2_ and 1.07 ± 0.03% in the ChCl–urea–ZnCl_2_–CeCl_3_ liquid.

### 2.2. Electrochemical Characterization

Cyclic voltammetries were performed using a conventional three-electrode cell. For platinum substrate, screen-printed electrodes (SPE) (DropSens S.L, Llanera, Asturias, Spain) comprised by platinum, platinum and silver were used as working, counter and pseudo-reference electrodes respectively. The area of the working electrode is 0.126 cm^2^. Carbon steel substrate was used as working electrode with an exposed area of 0.313 cm^2^, a platinum wire as counter electrode and a silver was used as wire as pseudo-reference electrode.

The voltammograms were performed from −2 V to +1.5 V at a scan rate of 10 mV/s. Potential sweep started cathodically at open circuit potential up to −2 V. Then, at this voltage the reverse scan started and the anodic voltage sweep was carried out, up to 1.5 V.

### 2.3. Hull Cell Test

Hull cell test allows obtaining, in one sample, electrodeposited alloys with a wide variety of surface morphologies and chemical compositions as result of the different current densities. The electrodeposition of Zn–Ce coatings was carried out by using a standard 267 mL Hull cell (Kocour Company, Chicago, IL, USA). A Hull cell it is a trapezoidal cell with and inclined cathode respect to the anode and therefore there is distribution of current density along the cathode that depends on the total current and the distance of each point of the cathode to the nearest point of the cathode to the anode [46]. In this manner identification of optimum current density ranges can be made from a single test. A platinum coated titanium plate and a carbon steel plate were used as anode and cathode, respectively. Prior to electrodeposition, the steel plate was polished with 180 grain SiC sandpaper and then degreased by methyl-ethyl-ketone (MEK) (Merck KGaA, Darmstadt, Germany). Finally, the samples were rinsed with deionized water. Before electrodeposition, the steel samples were chemically pickled for 1 min in hydrochloric acid solution, HCl 35% vol. containing 3.5 g/L of Hexametil Ethylene Tetramine (Merck KGaA, Darmstadt, Germany) as corrosion inhibitor. 

The electrodeposition was performed at 80 °C at a constant applied current of 0.25 A for 60 min, which produced cathode current densities over the steel plate ranging from 2.58 to 0.01 A/dm^2^, approximately [47].

### 2.4. Corrosion Resistance and Coating Behaviour

Electrochemical behaviour of the electrodeposited Zn–Ce coating and the Zn coating used as reference was evaluated by means of potentiodynamic polarization curves using a conventional three-electrode cell with a GAMRY reference 600 potentiostat. (Gamry Instruments, Warminster, PA, USA) An Ag/AgCl (3 M KCl) electrode was used as reference electrode, a platinum wire as the counter electrode and an exposed area of 0.313 cm^2^ of the electro-coated steel under study as the working electrode. The solution used as electrolyte was 0.05 M of NaCl. Open Circuit Potential (OCP) was recorded during 24 h of immersion in a 0.05 M NaCl solution and afterwards cathodic polarization curves were carried out. The potential sweep started anodically from 300 mV bellow OCP at a scan rate of 0.16 mV/s.

Furthermore, in order to corroborate the active corrosion protection of cerium, scratched Zn and Zn–Ce coated samples were immersed in a 0.05 M NaCl solution for 5 h. A coating/substrate area ratio of 15:1 was imposed in order to mimic a realistic situation based on the most common types of damages occurred in service, such as scratches and pinholes, i.e., a large anode and small cathode.

### 2.5. Structure and Chemical Composition of the Coatings 

Microstructural characterization and chemical composition of the Zn–Ce coatings was performed by a Scanning Electron Microscopy (SEM) (S-4800J, Hitachi Ltd., Tokyo, Japan) equipped with Energy Dispersive X-ray spectroscopy (EDX).

X-Ray Photoelectron Spectroscopy (XPS) spectra were measured in an ultrahigh vacuum system at a base pressure below 1 × 10^−9^ mbar using a hemispherical analyzer (Phoibos 100 MCD-5, SPECS Surface Nano Analysis GmbH, Berlin, Germany). The pass energy was 9 eV, giving a constant resolution of 0.9 eV. The Au 4f7/2, Ag 3d5/2 and Cu 2p3/2 lines of reference samples at 84.0, 368.3 and 932.7 eV, respectively, were used to calibrate binding energies. A twin anode (Mg and Al) X-ray source was operated at a constant power of 300 W using Mg Kα radiation (hυ = 1253.6 eV). All XPS spectra shown below are at the same scale, therefore intensity of the peaks are proportional to the amount of Cerium in the different zones.

## 3. Results and Discussion

### 3.1. Cyclic Voltammetry (CV) Study of the Ionic Liquid with Zn, Ce, and Zn–Ce Salts

**Figure 1:**
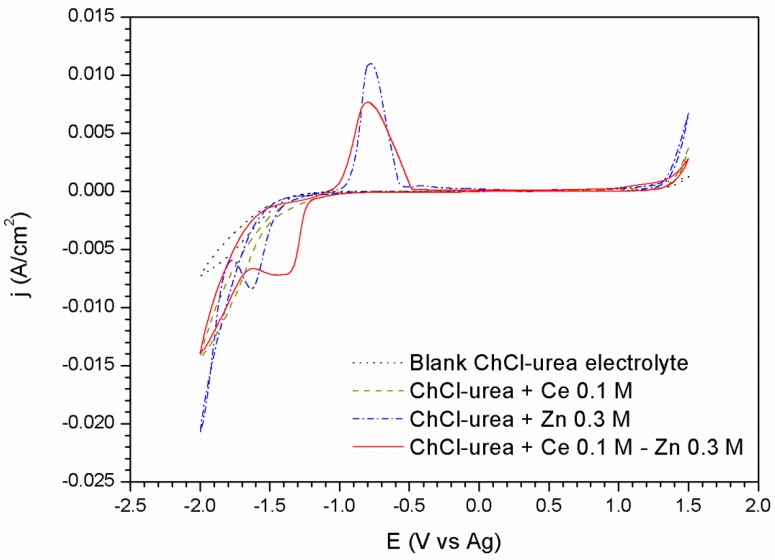
Voltammograms corresponding to the platinum substrate: in the blank ionic liquid, with 0.3 M of Zn salt, and with 0.3 M of Zn salt and 0.1 M of Cerium salt.

**Figure 2:**
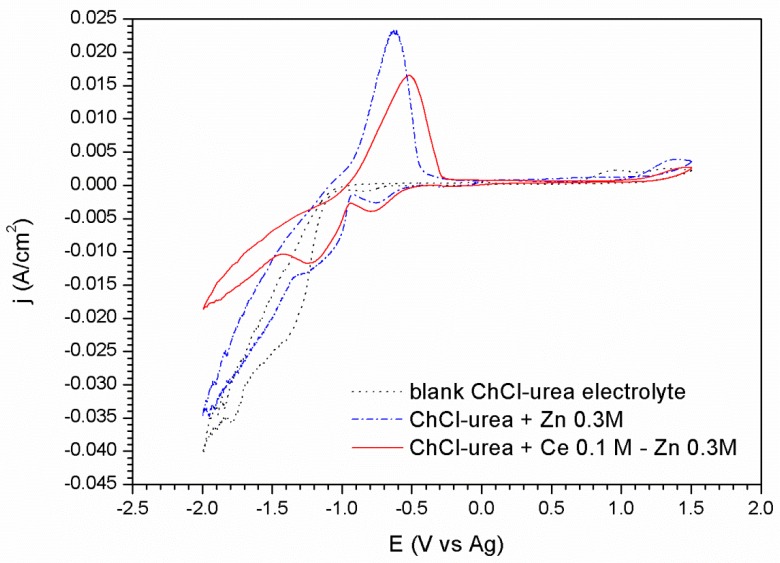
Voltammograms corresponding to the steel substrate: in the blank ionic liquid, with 0.3 M of Zn salt, and with 0.3 M of Zn salt and 0.1 M of Cerium salt.

Oxidation/reduction processes of the Zn and Ce metal salts in the ChCl/Urea ionic liquid have been studied by cyclic voltammetry for both platinum and carbon steel substrate, Figure 1 and Figure 2, respectively.

The voltammogram corresponding to the platinum substrate in the ionic liquid without the presence of the metallic salts (Figure 1) shows its window of electrochemical stability, without any oxidation peak up to +1.3 V, and the corresponding reduction peak described in the cathodic sweep at −1.4 V, associated with the decomposition of the ionic liquid and a possible evolution of the water present in the DES. 

The voltammogram corresponding to the platinum substrate in an electrolyte composed of DES and a concentration of 0.3 M ZnCl_2_ (Figure 1), shows a reduction peak at −1.63 V related to the reduction reaction of Zn^2+^ to Zn^0^ species, as demonstrated in later sections. The subsequent increase of the current density from the −1.79 V potential, is associated with the decomposition of the medium together with the deposition of Zn. In the reverse sweep, the formation of a cross-over at a potential of −1.73 V suggests the existence of a nucleation overpotential attributed to the existence of a new phase on the substrate. This nucleation overpotential is due to the fact that the electrodeposition of Zn requires more negative potentials to be carried out at the zinc/platinum interface than at the zinc/zinc. On the other hand, the anodic scan shows a single peak at −0.75 V associated with the oxidation of the electrodeposited Zn coating. The overlapping of the ionic liquid decomposition and the Zn reduction reactions prevents to confirm the reversible charge transfer of the reduction/oxidation of zinc by comparing the areas of the reduction and oxidation peaks of Zn.

The incorporation of Ce^3+^ in the ChCl–Urea DES does not seem to alter the voltammogram respecting to the blank electrolyte. However the incorporation of Ce^3+^ ions in the ZnCl_2_–ChCl/Urea system (Figure 1) clearly influences on the zinc reduction reaction. A wider peak shifted towards less negative values can be observed at −1.38 V followed by a current plateau extending up to −1.60 V. Thus, no individual reduction peaks for each specie is observed, but the joint response of both. For a potential of −1.65 V the beginning of a second reduction process is observed comprised by the degradation process of the DES.

For the steel substrate the appearance of the voltammograms changes (Figure 2). The curve corresponding to the ionic liquid without metallic salts shows a similar window of electrochemical stability. This voltammogram is comprised by two reduction peaks described in the cathodic sweep. The first one located at −0.83 V appears as a small shoulder and it is related to the decomposition of the choline cation [48]. It is worthwhile to mention that this process is not observed in the platinum substrate. Secondly, a steep increase of the cathodic current takes place at −1.07 V. This increase is attributed to the formation of hydrogen as result of the urea reduction reaction according to the literature [49,50,51] as well as a possible reduction of the water present in the ionic liquid.

The addition of the species ZnCl_2_ in the electrolyte (Figure 2) promotes clear changes in the voltammograms. Three reduction processes are clearly revealed in the cathodic sweep. The first peak related to reduction of the choline cation appears slightly shifted towards less negative potential values, −0.75 V, in comparison to the electrolyte free of metallic species. A second peak related to the massive reduction of Zn^2+^ to Zn^0^ starts at −0.93 V. Finally, at −1.35 V, an abrupt increase of the cathodic current points out the decomposition of the medium together with Zn reduction. During the anodic scan, a high intensity appears at −0.62 V, related to the oxidation of the electrodeposited Zn.

The voltammogram corresponding to the ionic liquid containing 0.3 M of ZnCl_2_ and 0.1 M of CeCl_3_·7H_2_O (Figure 2), shows a similar response than in ChCl/Urea + ZnCl_2_ 0.3 M electrolyte. The first peak corresponding to the reduction reaction of the choline cation at –0.75 V shows higher intensity immediately after this process, an increase in the current density leading to the formation of a peak centered at −1.23 V is observed. This second peak, appearing now more clearly resolved likely corresponds to the reduction of zinc modified by the presence of Ce species. The reduction wave which begins at −1.45 V is due to the co-deposition of Zn and the decomposition of the medium although in this case the presence of cerium causes this process to have a lower current density than those associated with the other cases. As it is well known, the deposition mechanism of cerium compounds is not electrochemical but chemical. This fact is observed in the voltammograms by the modification of the peaks attributed to the reduction/oxidation processes of the zinc and of the other species in the medium; but also by the precipitation of cerium oxides as a result of their nature as a cathodic inhibitor. These Ce precipitates block the cathodic reaction leading to lower current densities (Figure 1 and Figure 2).

### 3.2. Characterization of Hull Cell Samples

**Figure 3:**
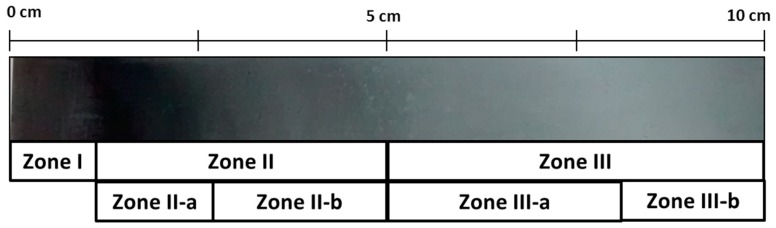
Macrophotography of a Hull cell specimen obtained in 0.3 M ZnCl_2_–0.1 M CeCl_3_–ChCl–Urea with the different color zones indicated.

**Figure 4:**
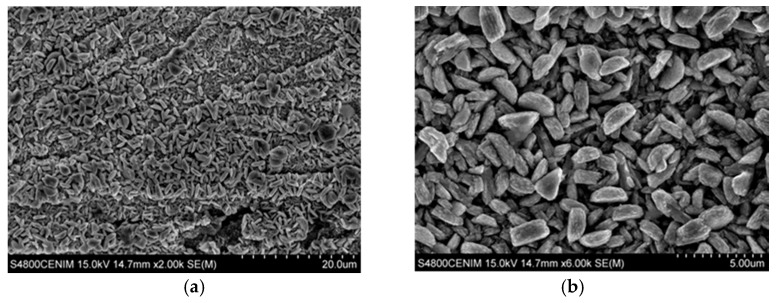

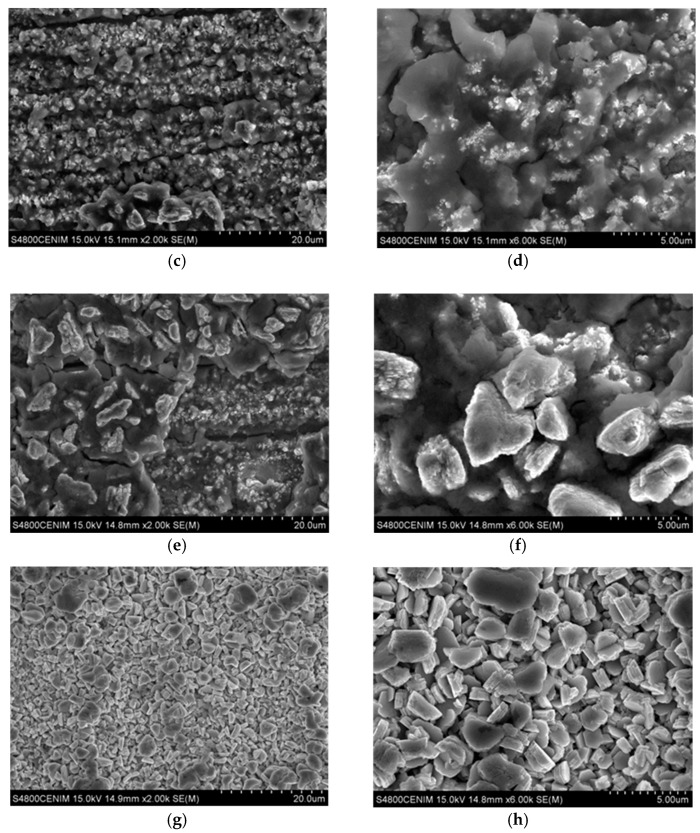
Scanning electron microscopy (SEM) images taken at different position of the Hull cell specimen obtained in 0.3 M ZnCl_2_–0.1 M CeCl_3_–ChCl–Urea: (**a**) dark gray area (zone I); ×2000; (**b**) ×6000; (**c**) Intense dark blue area (zone II-a), ×2000; (**d**) ×6000; (**e**) dark blue area (zone II-b) ×2000; (**f**) ×6000; (**g**) light gray area (zone III) ×2000; and (**h**) ×6000.

**Figure 5:**
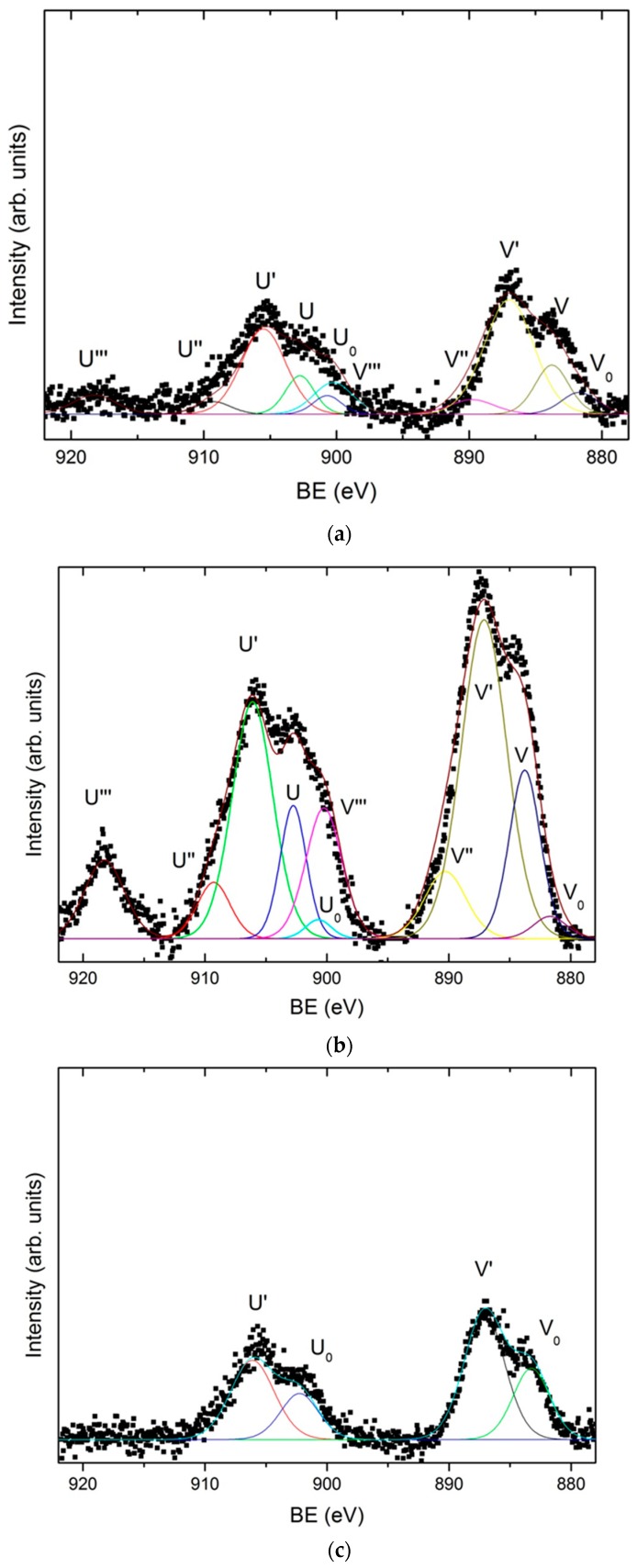

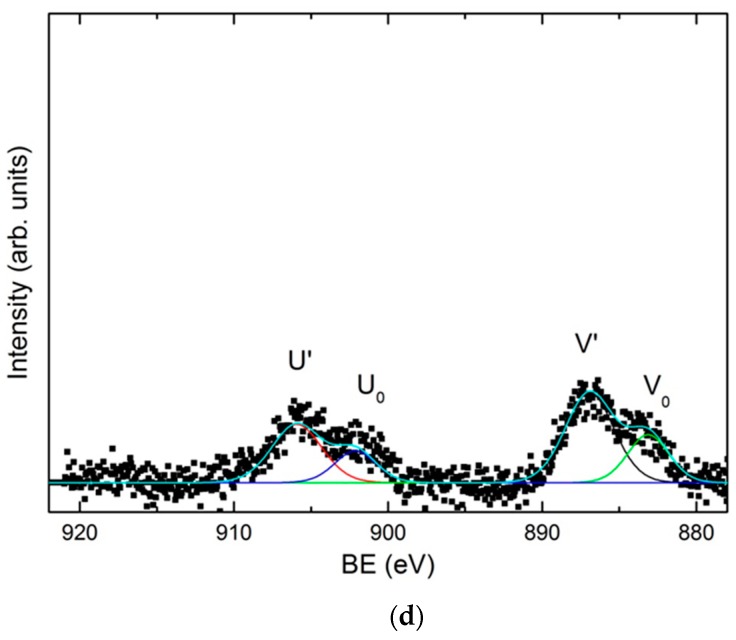
High-resolution X-ray photoelectron spectroscopy (XPS) spectrum corresponding to Ce 3d for: (**a**) dark gray area (zone I); (**b**) dark blue area (zone II-a); (**c**) dark blue area (zone II-b); and (**d**) light gray area (high current density extreme of zone III-a).

Zn coatings were further studied by cathodic electrodeposition using a Hull cell on carbon steel, from an electrolyte of ChCl–Urea ionic liquid with the respective concentrations of zinc chloride and cerium. The macrograph gathered in Figure 3 shows the appearance of the sample produced in the hull cell. As it can be seen at different positions of the sample (i.e. at different current densities during deposition) three different coloring zones can be distinguished. Henceforth the following notation based in colors appreciated visually will be used to identify the morphological features (Figure 4) and compositional studies carried out by SEM, EDX, and XPS (Figure 5 and Table 1) on each zone: dark gray (Zone I), dark blue (Zone II) and light gray (Zone III). Table 1 compiles and correlates all the results of the surface chemical composition of the electrodeposit obtained by EDX as a function of the current density for each zone studied.

The results show that the dark gray area (Zone I) corresponds to the deposition performed at the highest current densities, with values comprised between 2.59 and 1.22 A/dm^2^. The surface morphology observed by SEM in the dark grey area (zone I), Figure 4a,b shows the presence of a homogenous coating made of crystals randomly oriented. At higher magnifications these particles reveal nano-laminated features. According to the EDX data, this area has a homogeneous chemical composition with an average content of Zn, Ce, and O of 81.73 ± 1.90 at. %, 1.26 ± 0.34 at. %, and 17.01 ± 1.79 at. %, respectively. X-ray diffraction results (Supplementary Material S1) showed that zinc is electrodeposited in this zone as metallic zinc. On the other hand, the results obtained by XPS confirm the presence of cerium as a mixed oxide with a composition of 52.2 % Ce_2_O_3_–47.7 % CeO_2_ (Figure 5a). As can be observed in Figure 5a, the deconvolution of the high resolution spectrum of the Ce 3d region, after linear background subtraction, shows up to 10 peaks. The complexity of the cerium spectrum is due to the coexistence of two different cerium oxidation states, Ce (IV) and Ce (III), as well as the effects of covalent hybridization and spin-orbit splitting [52,53,54]. The presence of Ce (IV) is noted by the doublets associated to a bonding energy of 883.8 eV/902.8 eV, 890.3 eV/909.3 eV, and 900.3 eV/918.3 eV, commonly denominated v–u, vII–uII and vIII–uIII, respectively. Of special significance is the satellite peak uIII which is characteristic of Ce (IV). On the other hand, the existence of Ce (III) is associated with doublets at 883.1 eV/902.1 eV and 886.9 eV/905.9 eV, peaks usually named vo–uo and vI–uI, respectively.

The dark blue area (zone II), delimited between current density values of 1.13 and 0.36 A/dm^2^ can be further divided in two subzone according to the SEM and EDX measurements. In the first subzone (Zone II-a), of a more intense dark blue, with the electrodeposition current density range comprised from 1.13 to 0.75 A/dm^2^, EDX data shows the maximum contents of cerium and oxygen, 8.30 ± 1.36 at. % and 55.31 ± 1.72 at. % in average, respectively; and the minimum content of zinc, 36.39 ± 2.20 at. %. The SEM images for this zone II-a (Figure 4c,d) show that the zinc crystals (the brightest particles) are embedded in an amorphous-like structure mainly composed of cerium oxides. Such zinc particles apparently are smaller in size than those observed in Zone I and some cracks and pores can be also observed within the coating. XPS shows that cerium is present as a mixture of oxides (50.6 % Ce_2_O_3_–49.4 % CeO_2_), Figure 5b. Nevertheless, for this range of current densities, a higher amount of cerium mixed oxide is deposited, with a slightly increasing presence of CeO_2_ in comparison to the zone electrodeposited at higher current densities (zone I).

The second dark blue subzone (Zone II-b) corresponds to current densities ranging from 0.75 to 0.36 A/dm^2^. The color of this zone turns from dark blue to greener and lighter shades. This variation of color corresponds, according to XPS data, to a concentration gradient of the elements in the electrodeposited coating. The content of cerium and oxygen varies directly proportional to the current density according EDX data, from 8.89 to 4.14 at. % for cerium and from 50.08 to 19.14 at. % for oxygen. These results show that the Ce/O ratio is constant regardless of the current density. Meanwhile zinc content increases from 41.03 to 76.71 at. %, verifying that Zn/Ce and Zn/O ratios are inversely proportional to the current density. XPS analysis revealed that in this sub-zone II-b, cerium oxide is essentially Ce_2_O_3_, Figure 5c. The Ce 3d spectrum, Figure 5c, shows only the doublets associated with Ce (III), vo–uo y vI–uI, at binding energies of 883.1 eV/902.1 eV and 886.9 eV/905.9 eV, respectively. These results suggest that for current densities ranging from 0.75 to 0.36 A/dm², the cathodic processes do not generate enough OH^-^ ions to induce the local pH increase required for the formation of Ce (IV) species. The surface morphology of this zone is shown in Figure 4e,f. Although similar to the zone II-a previously discussed, it is worthwhile to highlight that larger zinc particles are embedded in the cerium oxide matrix. That is, it appears that lower current densities promote larger size of zinc particles.

Finally, the light gray zone (Zone III) corresponds to values of current density lower than 0.36 A/dm^2^. According to EDX data the average content of Zn and oxygen is 90.91 ± 2.13 at. % and 8.75 ± 1.88 at. %, respectively. Meanwhile, the content of cerium deposited in these conditions is very low, of about 0.54 ± 0.22 at. % for 0.36 A/dm^2^ to be negligible for densities lower than 0.13 A/dm^2^ (Zone III-b). The XPS spectrum shown in Figure 5d indicates that cerium is still present, as Ce_2_O_3_, but only at high current density current zone, being undetectable as current density decreases in the Hull cell sample. The SEM analysis of this area, shows that practically only the deposition of zinc takes place, consequently the surface morphologies obtained present the characteristic electrodeposition of zinc (Figure 4g,h), where the particles form a homogeneous, compact and uniform coating with micro–crystals of hexagonal morphologies, similar to those reported in literature for Zn electrodeposition from ionic liquids [7,55,56]. These crystals appear to be larger at lower current density area of the hull sample.

In general, it can be concluded that zinc is deposited in metallic state according X-Ray Diffraction XRD (Supplementary Material Figure S1) over the entire range of current densities studied. Conversely, according to XPS data cerium is always deposited in form of oxide as result of their chemical precipitation process. Nevertheless, the type of oxide deposited appears to be influenced by the current density. At lower current densities, of about 0.13 A/dm^2^, cerium precipitates as cerium (III) oxide, Ce_2_O_3_, while for higher values than 0.36 A/dm^2^ cerium precipitates as a mixture of Ce_2_O_3_ and CeO_2_ oxides. The maximum proportion of the cerium (IV) oxide CeO_2_ appears in the zone IIa, electroplated with a current density ranging between 1.13 and 0.75 A/dm^2^. Due to the fact that a small quantity of water is always present in the different ChCl–urea mediums used for the electrodeposition [56] within the Zn–Ce coatings Ce precipitation in form of oxide species can be interpreted by the mechanism discussed in the literature for aqueous media. First, the cathodic current produces the generation of OH^-^ ions by the reduction of the H_2_O present in the electrolyte. This cathodic electrogeneration of OH^-^ ions produces a localized increase in pH in the cathodic zones, thermodynamically allowing the chemical precipitation of cerium oxides and hydroxides on the surface by the reaction of the Ce^3+^ ions with the OH^-^ ions. Furthermore the presence of cerium (IV) species appears to be formed by the direct oxidation of Ce^3+^, or through the formation via oxidation of an intermediate ionic soluble complex and its subsequent precipitation [19]. Other works have proposed that CeO_2_ can be generated through a solid state reaction of Ce(OH)_3_ [57] or by oxidation and precipitation of Ce^3+^ by H_2_O_2_ generated by oxygen evolution [25,58]. In any case the higher presence of Cerium (IV) oxide in zone II-a indicates a higher pH or O_2_ concentration for those density currents [57,59].

**Figure 6:**
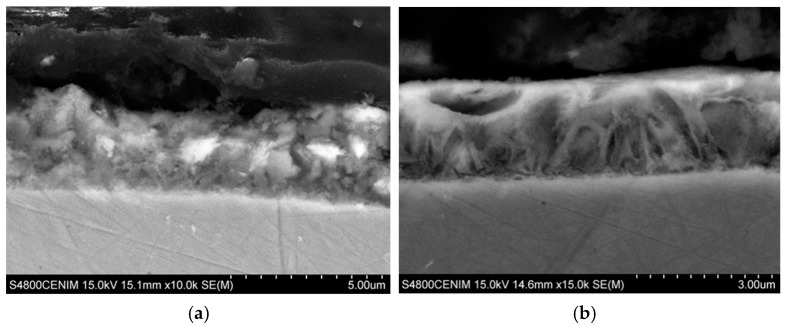
SEM images taken in cross sections at different position of the Hull cell specimen obtained in 0.3 M ZnCl2–0.1 M CeCl_3_–ChCl–Urea, at 0.25 A for 60 min: (**a**) Intense dark blue area (zone II-a) ×10,000 and (**b**) dark blue area (zone II-b) ×15,000.

Figure 6 shows the SEM images corresponding to the cross section of the coatings at zone II, where the cerium content is maximum. As it can be seen both coatings are homogeneous in thickness with no apparent porosity. Figure 6a shows the cross section of the coating grown in zone II-a with a powdery appearance, while the cross section of zone II-b shows a more ordered and compact structure, Figure 6b.

On the other hand, from the thickness of the cross section it can be seen that in these zone II deposition rate is in the range between 2–3 µm/h. A relatively slow deposition rate compared with the reported for Zn from ChCl–urea (5.5 µm/h) but comparable to the deposition rate when surfactants or brighteners are added to the ChCl–urea electrolyte [57].

### 3.3. Corrosion Resistance

The corrosion resistance of the electrodeposited Zn–Ce oxide coatings was evaluated in order to establish their corrosion kinetics, as well as their sacrificial properties and active protection in comparison with the zinc coated carbon steel.

**Figure 7:**
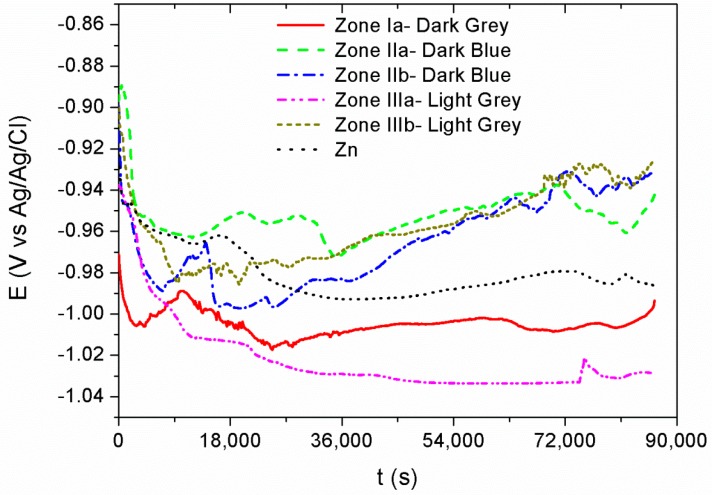
Open circuit potential (OCP) measurements recorded for 24 h for each of the differentiated areas—dark gray, dark blue, and light gray.

Figure 7 shows the open circuit potential (OCP) measurements recorded for 24 h for each of the previously differentiated areas—dark gray, dark blue, and light gray—according to the current densities of the Hull cell. In all cases, the potential decreases cathodically for five hours as a consequence of the reorganization of the charges on the metal surface at the surface-dissolution interface. After that time, the system reaches a steady state where OCP values remain relatively constant until the end of the test. All the registered potential values vary between −0.92 V and −1.03 V, typical values for a Zn coating in saline solution. Although previous studies reported shifts to noble potentials due to the effect of cerium on Zn–Ce intermetallic alloys in comparison with the Zn [58,59], in this work the Zn–Ce oxide coatings show similar OCP than Zn.

**Figure 8:**
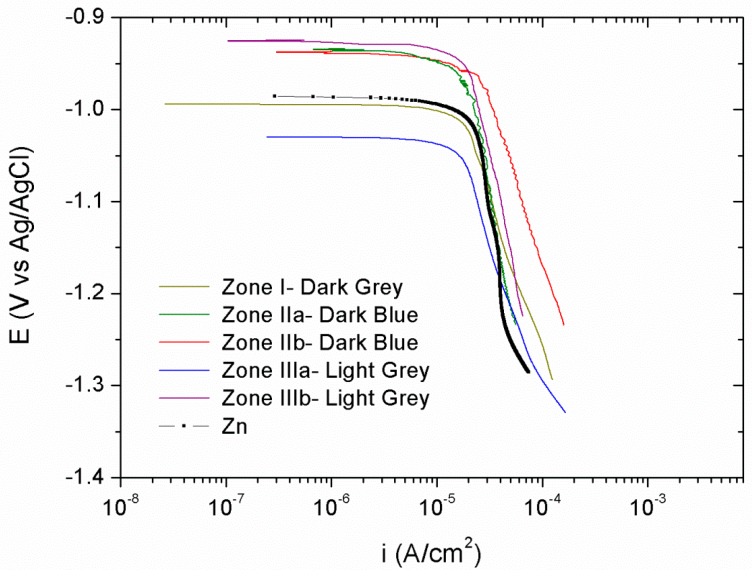
Cathodic polarization curves performed after 24 h of immersion in 0.05 M NaCl for each of the differentiated areas—dark gray, dark blue, and light gray.

Cathodic polarization curves, Figure 8, were performed after 24 h of immersion in 0.05 M NaCl. For comparison purposes the cathodic polarization curve for the electrodeposited Zn obtained at a current density of 1.3 A/dm^2^ (the average current density of all those generated in the Hull cell) was used as the reference. As it can be seen in Figure 8, no significant differences were observed among the curves obtained at different zones of the Hull sample with different morphologies and chemical compositions. All the cathodic polarization curves present the typical behavior of a diffusion control, and no significant differences were observed among the different Zn–Ce zones studied, and with respect to the pure Zn. The corrosion rate values (i_corr_) are presented in Table 2 together with the corrosion potential values (E_corr_). All the coatings reveal similar corrosion potentials and corrosion rate of about 10^−5^ A/cm^2^.

In this context, where there are hardly any differences in corrosion parameters between coatings of zinc and zinc–cerium, a more realistic test was carried out to evaluate the active protection role of the cerium contained in the electrodeposited coatings. This test consisted of making a scratch on the Zn and Zn/Ce_2_O_3_–CeO_2_ coatings, thus exposing the substrate. These defects in the coatings were made in the areas corresponding to a current density of 1.13 A/dm^2^, a zone in which a higher presence of cerium was recorded (Table 1). The scratch was performed maintaining a ratio of coating/substrate areas of 15:1, as this mimics realistic conditions under which this type of defect occurs in the coatings during its service [1]. After this, samples were immersed in a 0.05 M NaCl solution for 5 h and the corrosion products which developed on the surface of the substrate were studied using SEM and EDX.

**Figure 9:**
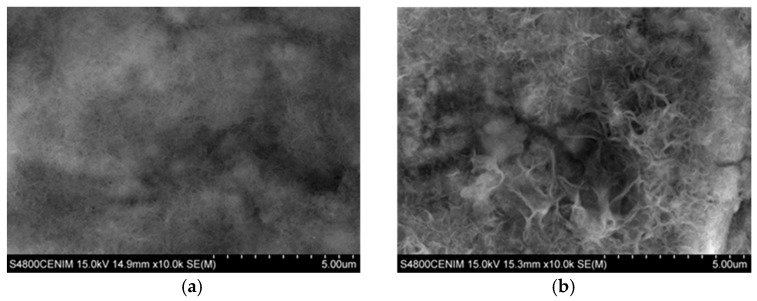
SEM image (×10,000) of the corrosion products generated on the scratch on the ratio area test 15:1: (**a**) Zn coating and (**b**) Zn/Ce_2_O_3_–CeO_2_ coating.

The results show that in the case of Zn coating (Figure 9a), after exposure to the corrosion medium, the scratched area was covered by corrosion products. EDX analysis reveals that these compounds are mostly zinc oxides/hydroxides (Table 3). This fact is because thermodynamically, the zinc acts as an anode with respect to the steel, Zn^2+^ ions can precipitate chemically when reacting with OH^-^ ions forming Zn(OH)_2_ and ZnO. However, although the literature indicates that this film causes the corrosion kinetics to be constant over a short potential range, it is also determined that this behavior is not attributed to having a protective effect, but to the fact that the formation of this layer is a process limited by mass transport [60,61]. In addition, depending on the release of zinc ions to the medium, and due to the fact that the zinc oxides/hydroxides can evolve slowly over time [62], other white rust corrosion products can be generated. These, depending on the medium, may be hydrozincite Zn_5_(CO_3_)_2_(OH)_6_, zinc hydroxysulfate Zn_4_SO_4_(OH)_6_·4H_2_O, or zinc hydroxychloride Zn_5_(OH)_8_Cl_2_·H_2_O, among others. However, they do not provide an optimum protection against corrosion due to their low adhesion [59], as well as their decreasing protective action with time, resulting of their evolution to less protective zinc soluble complexes- oxides [63].

For the Zn–Ce oxide coatings the morphology of the corrosion products on the scratched surface is similar to that of the Zn deposit. However, in this case it is observed that the fibers are larger and more defined as well as the presence of granular particles can be observed (Figure 9b). It should be noted that the analysis of the composition of these corrosion products by EDX (Table 3) shows the presence of zinc and cerium. The high content of oxygen appears to indicate that both are in oxide/hydroxide form. This fact therefore suggests that the cerium present in the coating as mixed oxide has migrated from the coating to the high pH cathodic areas developed on the surface of the steel substrate thus giving the coating some active protection role. 

The cerium oxides migration mechanism seems to be determined by the reduction of CeO_2_ to Ce_2_O_3_ during the anodic dissolution of zinc. The Ce_2_O_3_ oxide appears to be chemically soluble in NaCl solutions [25], thus producing the release of Ce^3+^ ions to the medium. These Ce^3+^ ions released into the medium develop a function as a cathodic inhibitor, reacting with the OH^-^ ions generated in the oxygen reduction cathodic reactions (occurring in the naked substrate), forming cerium oxides/hydroxides precipitates that block these cathodic areas, providing an active protection against corrosion. This mechanism is analogous to that developed during electrodeposition of the coating, as described in previous sections. On the other hand, during the anodic dissolution of zinc, the CeO_2_ particles can be released into the medium. It has been demonstrated that CeO_2_ present in an electrolyte can be transported to the cathode surface by a variety of factors; including electrophoresis, adsorption, convection, and diffusion, among others [64]. These CeO_2_ particles have a very high affinity for oxygen and charged ions such as Zn^2+^, and can precipitate forming Zn–Ce mixed corrosion stable products [62,65], which inhibit electrochemical corrosion reactions by polarizing anodic reactions, thus protecting the material.

Although the release and migration of the cerium present in the coating onto the surface of the cathodic substrate has been demonstrated, it is necessary to continue studying and developing these Zn-coatings with cerium oxides to optimize their active protective function.

## 4. Conclusions

It has been demonstrated for the first time that it is possible to electrodeposit coatings formed by nano-composite material with a cerium oxide reinforced metal zinc matrix, from a simple mixture of deep eutectic ionic liquids by the addition of zinc and cerium salts.

The morphology and composition varies with the current density of the electrodeposition, obtaining between 1.13 and 0.75 A/dm^2^ of the maximum values of the cerium amount of 8.30 ± 1.36 at. %, in the form of oxide 50.6% Ce_2_O_3_–49.4% CeO_2_. The cerium zinc coating shows an active migration of the cerium in case of defects in this coating.

## Figures and Tables

**Table 1:** Surface chemical composition of the electrodeposit obtained by EDX as a function of the current density for each zone.

Color	Zone	Measure	Zn (at. %)	Ce (at. %)	O (at. %)	Distance (cm)	Current Density (A/dm^2^)
**Dark grey**	**I**	1	82.76	1.01	16.24	0.1	2.59
		2	83.93	0.97	15.10	0.5	1.67
		3	81.59	1.24	17.17	0.7	1.48
		4	78.82	1.27	19.91	0.9	1.33
		5	81.56	1.83	16.61	1.1	1.22
**Dark Blue**	**II-a**	6	35.60	10.21	54.18	1.3	1.13
		7	34.11	9.11	56.78	1.6	1.01
		8	38.49	6.79	54.72	1.9	0.91
		9	38.95	7.63	53.42	2.2	0.83
		10	34.81	7.76	57.43	2.5	0.75
	**II-b**	11	41.03	8.89	50.08	2.7	0.71
		12	42.64	8.62	48.74	3.3	0.60
		13	60.69	5.36	33.94	3.9	0.50
		14	68.71	5.61	25.67	4.5	0.42
		15	76.71	4.14	19.14	5	0.36
**Light Grey**	**III-a**	16	89.76	0.52	9.72	5.1	0.35
		17	91.09	0.29	8.63	5.7	0.28
		18	90.61	0.43	8.96	6.3	0.23
		19	86.71	0.89	12.40	6.9	0.18
		20	91.87	0.56	7.56	7.5	0.13
	**III-b**	21	90.83	0.00	9.17	8.1	0.08
		22	94.03	0.00	5.97	8.7	0.04
		23	92.38	0.00	7.62	9.3	0.01

**Table 2:** Corrosion rate values (icorr) and corrosion potential values (Ecorr) obtained from polarisation curves.

Coating	Zone	OCP (V)	i_corr_ (A/cm^2^)	E_corr_ (V)
**Zn**	**General**	−0.98	2.29 × 10^−5^	−0.98
**Zn/Ce_2_O_3_–CeO_2_**	**I-Dark Grey**	−0.99	1.67 × 10^−5^	−0.99
	**II a-Dark Blue**	−0.94	1.78 × 10^−5^	−0.93
	**II b-Dark Blue**	−0.93	2.50 × 10^−5^	−0.93
	**III a-Light Grey**	−1.02	1.62 × 10^−5^	−1.02
	**III b-Light Grey**	−0.92	1.78 × 10^−5^	−0.92

**Table 3:** Surface chemical composition obtained by EDX of the corrosion products generated on the scratch on Zn and Zn/Ce_2_O_3_–CeO_2_ coatings.

Coating	Current Density (A/dm^2^)	Zn (at. %)	Ce (at. %)	O (at. %)
**Zn**	1.13	44.04 ± 2.29	-	55.96 ± 2.29
**Zn/Ce_2_O_3_–CeO_2_**		38.42 ± 3.36	3.28 ± 0.60	58.30 ± 3.26

## Supplementary Materials

The following are available online at www.mdpi.com/link. Figure S1: X-ray Diffraction spectra taken at different position of the Hull cell specimen obtained in 0.3 M ZnCl2–0.1 M CeCl3–ChCl–Urea: (a) dark gray area (zone I); (b) dark blue area (zone II); (c) light gray area (zone III).
