# Supplementary material for: Deposition of Zinc–Cerium Coatings from Deep Eutectic Ionic Liquids
Source: Materials (Basel). 2018 Oct 19;11(10):2035. doi: 10.3390/ma11102035 (PMC6213864; doi:10.3390/ma11102035)
Supplement: Supplementary file 1 [file materials-11-02035-s001.pdf]

materials

Article

Deposition of Zinc–Cerium Coatings from Deep Eutectic Ionic Liquids

Miguel Marín-Sánchez [1], Elena Gracia-Escosa [1], Ana Conde [1], Carlos Palacio [2] and Ignacio García [1,*]

[1] Department of Surface Engineering, Corrosion and Durability, National Center for Metallurgical Research CENIM-CSIC, Av. Gregorio del Amo 8, 28040 Madrid, Spain; mgl.marin.sanchez@gmail.com (M.M.-S.); graciaesc@gmail.com (E.G.-E.); a.conde@cenim.csic.es (A.C.)

[2] Department of Applied Physics, College of Science, Module 12, Autonomous University of Madrid, Cantoblanco, 28049 Madrid, Spain; carlos.palacio@uam.es

* Correspondence: igarcia@cenim.csic.es; Tel.: +34-915538900

Received: 01 October 2018; Accepted: 17 October 2018; Published: date

Supplementary Material

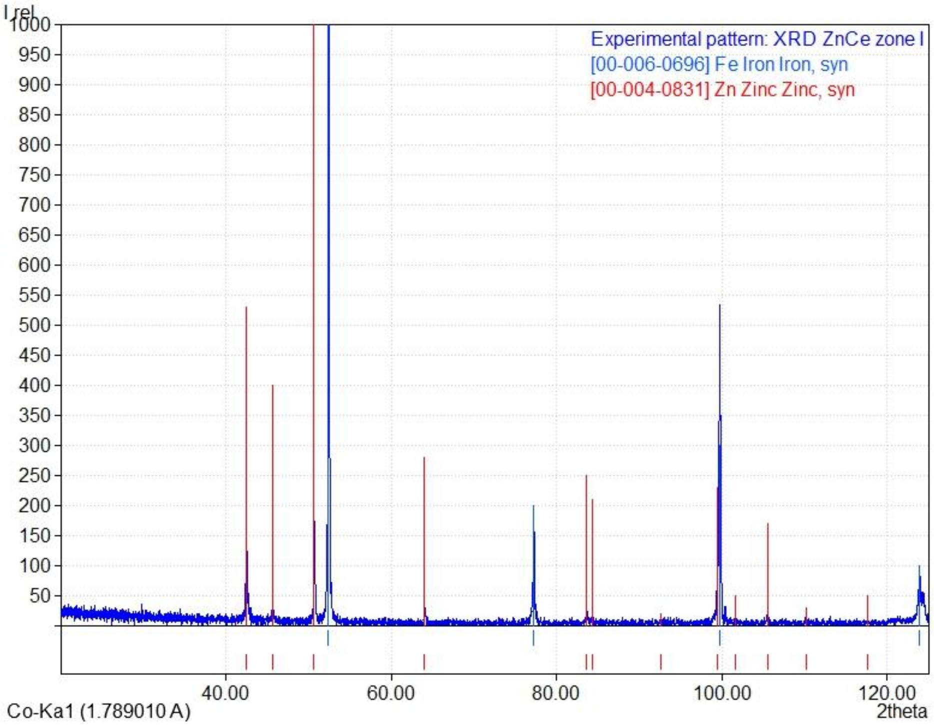

(**a**)

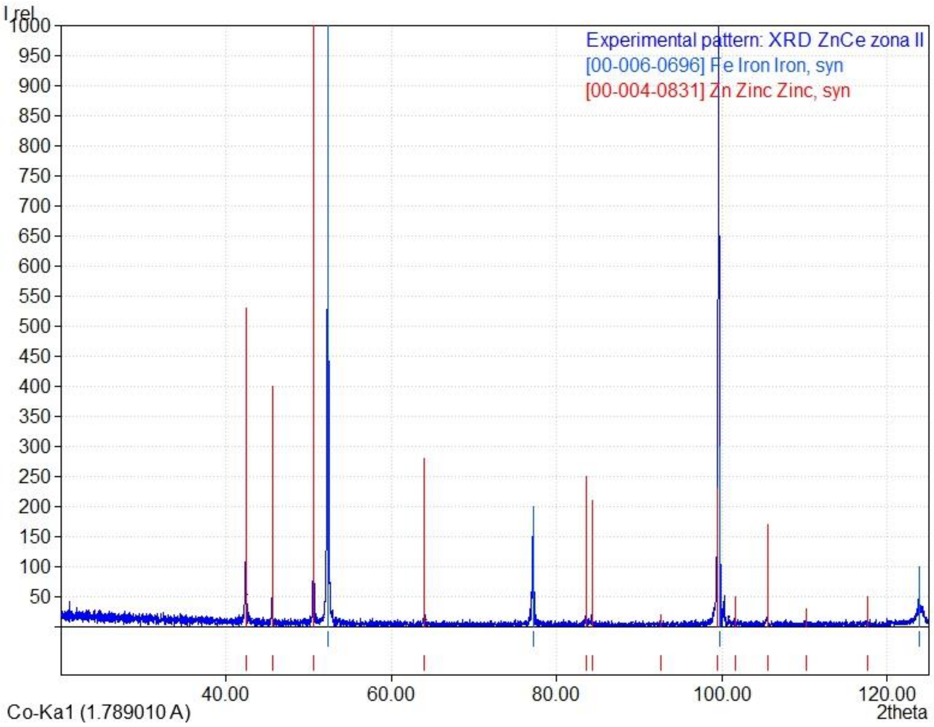

(**b**)

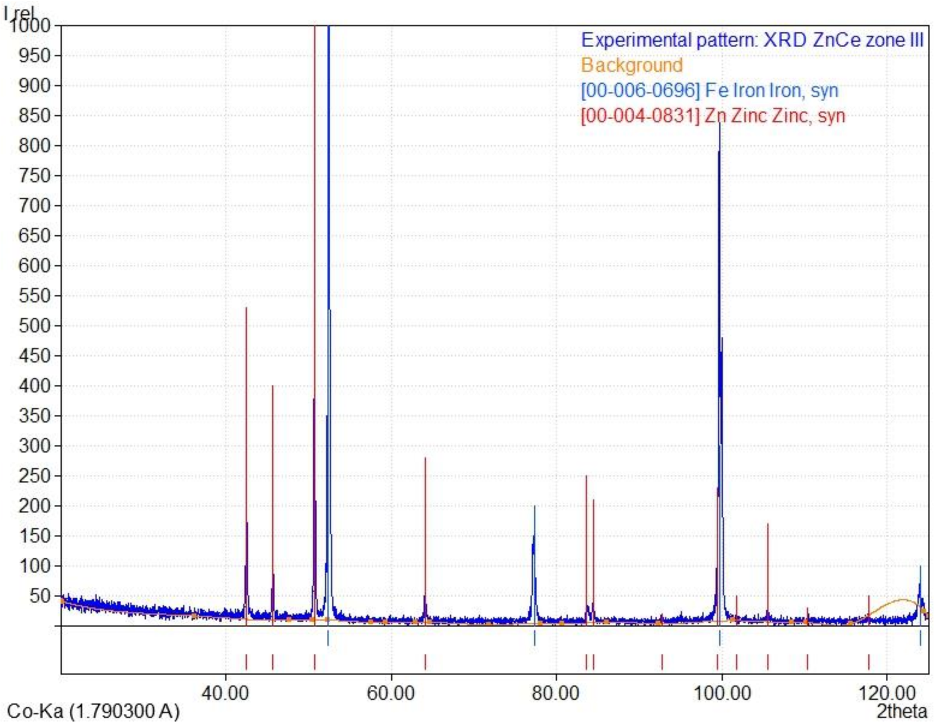

(**c**)

Figure S1. X-Ray Diffraction spectra taken at different position of the Hull cell specimen obtained in 0.3 M ZnCl2–0.1 M CeCl3–ChCl–Urea: (a) dark gray area (zone I); (b) dark blue area (zone II); (c) light gray area (zone III).

© 2018 by the authors. Submitted for possible open access publication under the terms and conditions of the Creative Commons Attribution (CC BY) license (http://creativecommons.org/licenses/by/4.0/).
